# The risk of injuries during work and its association with precipitation: New insight from a sentinel-based surveillance and a case-crossover design

**DOI:** 10.3389/fpubh.2023.1117948

**Published:** 2023-03-02

**Authors:** Tian Tian, Xiao Lin, Tingyuan Huang, Kai Zhang, Congxing Shi, Pengyu Wang, Shimin Chen, Tong Guo, Zhiqiang Li, Pengzhe Qin, Boheng Liang, Wangjian Zhang, Yuantao Hao

**Affiliations:** ^1^Guangdong Key Laboratory of Medicine, Department of Medical Statistics, Center for Health Information Research, School of Public Health, Sun Yat-sen University, Guangzhou, Guangdong, China; ^2^Guangzhou Center for Disease Control and Prevention, Guangzhou, Guangdong, China; ^3^Department of Environmental Health Sciences, University at Albany, The State University of New York, Rensselaer, NY, United States; ^4^Sun Yat-sen Global Health Institute, Sun Yat-sen University, Guangzhou, Guangdong, China; ^5^Peking University Center for Public Health and Epidemic Preparedness and Response, Peking University, Beijing, China

**Keywords:** precipitation, work-related injury, susceptibility, case-crossover, sentinel-surveillance

## Abstract

**Background:**

Injuries during work are often exogenous and can be easily influenced by environmental factors, especially weather conditions. Precipitation, a crucial weather factor, has been linked to unintentional injuries, yet evidence of its effect on work-related injuries is limited. Therefore, we aimed to clarify the impact of precipitation on injuries during work as well as its variation across numerous vulnerability features.

**Methods:**

Records on the work-related injury during 2016–2020 were obtained from four sentinel hospitals in Guangzhou, China, and were matched with the daily weather data during the same period. We applied a time-stratified case-crossover design followed by a conditional logistic regression to evaluate the association between precipitation and work-related injuries. Covariates included wind speed, sunlight, temperature, *SO*_2_, *NO*_2_, and *PM*_2.5_. Results were also stratified by multiple factors to identify the most vulnerable subgroups.

**Results:**

Daily precipitation was a positive predictor of work-related injuries, with each 10 mm increase in precipitation being associated with an increase of 1.57% in the rate of injuries on the same day and 1.47–1.14% increase of injuries on subsequent 3 days. The results revealed that precipitation had a higher effect on work-related injuries in winter (4.92%; 95%CI: 1.77–8.17%). The elderly (2.07%; 95%CI: 0.64–3.51%), male (1.81%; 95%CI: 0.96–2.66%) workers or those with lower educational levels (2.58%; 95%CI: 1.59–3.54%) were more likely to suffer from injuries on rainy days. There was a higher risk for work-related injuries caused by falls (2.63%; 95%CI: 0.78–4.52%) or the use of glass products (1.75%; 95%CI: 0.49–3.02%) on rainy days.

**Conclusions:**

Precipitation was a prominent risk factor for work-related injury, and its adverse effect might endure for 3 days. Certain sub-groups of workers were more vulnerable to injuries in the rain.

## 1. Introduction

Work-related injury is recognized to impose a significant health risk on workers worldwide. In 2019, the International Labor Organization (ILO) estimated that approximately 380,000 fatal occupational injuries and 374 million nonfatal occupational injuries occur each year, resulting in about 10.5 million disability-adjusted life years (DALYs) globally (1). Across the global landscape of worker health research, injuries are unique in that they are almost universally avertable and yet can still cause death or disability at any time. Even occupational injuries as elemental as falls from high could lead to devastating consequences such as spinal cord injuries, which may cause lifelong disability and contribute major costs to healthcare systems and societies (2). In addition to causing suffering and disability, the shorter term injuries for workers, such as an arm fracture can cause loss of human capital. The direct global economic loss from employee disablement and absenteeism has been estimated to be almost 4% of the world's Gross Domestic Product (GDP) (3). As a result, occupational injuries are recognized as being a source of lost health, human capital, and economy that could be averted with improved safety and prevention programs and ensuring access to care resources. Evaluating various risk factors that contribute to occupational injuries is crucial to reducing occupational injuries, and improving the social productivity and economy.

Numerous studies have shown that some individual factors (e.g., being stressed, low level of literacy and haste) and certain job features (e.g., defective or dangerous machinery, poor lighting conditions, and poor training procedures) were potential risk factors for injuries at work (4–7). Studies done in Australia, Qatar and Iran indicated disparities in the risk of injury across age, gender and occupation subgroups, suggesting a higher risk of work-related injuries among older workers, males and construction industry workers (8–10). Other studies reported that struck by object was the commonest cause of work-related injury and upper limb was the commonest body part involved (11). These findings suggest that stratified subgroup analysis based on worker characteristics may be necessary to fully explore the risk factors for work-related injuries.

Recent studies also suggested some environmental factors as potential risk factors for injuries, including meteorological factors (12, 13) and air pollution (14). Precipitation is among the most important environmental factors which were suggested to be associated with the increased risk of total injury (15, 16). For example, in the United States, according to the hospital records during 1996–2002, each inch of precipitation was associated with an increase of 68–70% in trauma admissions (17). The underlying mechanism may include the hasty behaviors of residents, lower visibility and unsafe traffic conditions such as slippery roads which were more likely to occur on rainy days (16). However, existing studies were usually based on a single center's hospital admission data. The admission data may not be able to capture the whole picture of injuries occurring in the region since many cases were not as severely injured as to be hospitalized (15, 16). Therefore, admission data may only catch the tip of iceberg, whereas the majority would go to outpatient and emergency department. A larger data based on outpatient and emergency department visits would be necessary to reveal a more accurate situation. Furthermore, existing studies usually were focused on general injuries or those related to driving, football league or steeplechase (18, 19). No studies, however, have investigated the effect of precipitation on injuries during work. Lastly, although several previous studies have suggested the vulnerability of certain populations toward the adverse impact of weather (20, 21), the effect of precipitation on different subgroups of occupational injuries remains unclear, hindering the development and implementation of targeted public health prevention and control strategies.

This study aims to quantify the effect of precipitation on work-related injuries and determine the effect disparity across different subgroups. Findings from this study may inform evidence-based decision-making targeted on the effect of precipitation by improving the working conditions and facility performance, and promoting health education programs, especially among the most vulnerable groups.

## 2. Materials and methods

### 2.1. Study population and injury data

Data regarding each case of work-related injuries in Guangzhou, China during 2016–2020 were obtained from the outpatient and emergency department of four injury-sentinel-surveillance hospitals in the city. The four monitoring sites are distributed in different districts, covering different types and levels of hospitals, with strict quality control systems and low underreporting rates (22). Attending doctors at surveillance hospitals collected the information on outpatients initially diagnosed with a work-related injury and reported it to the surveillance system. In this study, work-related injury was defined as an injury that occurred during the course of employment (23), while considering the respective situations involving both indoors and outdoors. The chance of injuries occurred indoors may be elevated due to the slippery and humid conditions brought by rainy weather. For example, previous studies have shown that, as a common phenomenon in the study area, the surface of indoor objects/floors usually gets wet and slippery in rainy and humid weather conditions (24). For the purpose of this study, participants who were injured at the time of working and between the ages of 18 and 65 years were included, and participants with injuries at home were excluded. All of the subjects were diagnosed with unintentional injuries [the International Statistical Classification of Diseases and Related Health Problems 10th Revision (ICD-10) codes: V01-Y89] caused by falls, traffic accidents, blunt injuries, sprains, or penetrating injuries during work. Furthermore, we collected information on the feature of injury, including the location and the cause of the injury, the sociodemographic information including sex (male or female), age groups (18–44 or 45–64), educational level (middle school and below or high school and above), occupation (agricultural/technicians, commercial/service or production/transport equipment operators), household register (provincial domicile, city domicile or out of province) and date of the injury. We also retrieved clinical information about the injury, including categories of the injury (such as contusion, fracture, internal organ injury, and penetrating injury), severity, affected body part and system involved. Participants whose admission outcome were “leave after treatment” or “observation patients” were defined as moderate injury cases, while those who were “hospitalized”, “transferred” or “dead” were defined as severe cases.

### 2.2. Air pollution and weather data

Daily average meteorological data, including ambient temperature (°C), precipitation (mm), wind speed (m/s) and hours of sunlight (h), were obtained from the China Meteorological Data Sharing Service System (https://data.cma.cn). According to the location of the sentinel hospital, we assigned the meteorological data from the nearest monitoring station (site number: 59287) on the same day to injury cases. Daily station-level air pollution data for Guangzhou during 2016–2020 were collected from the China General Environmental Monitoring Station (http://www.cnemc.cn). Upon the correlation test, Sulfur dioxide (*SO*_2_), nitrogen dioxide (*NO*_2_) and particulate matter (*PM*) ≤ 2.5 μm aerodynamic diameter (*PM*_2.5_) were selected as covariates for the model. We also assigned the daily average contamination concentration of the nearest monitoring site to the sentinel hospital in order to account for the daily exposure of each injury case.

### 2.3. Study design and statistical analyses

We performed a time-stratified case-crossover design which has been widely applied in environmental epidemiological studies (25, 26). For each case, the day of injury occurrence was defined as a case day while other days of the same weekday within the same calendar month were defined as control days. Since each case was served as its own control in this design, the potential confounders such as age, sex, occupation, education and socioeconomic status of workers would be automatically adjusted by design (27, 28).

Since all the variables representing social-demographic status or the features of injury were categorical, we described these variables by frequencies and percentages, in total, and by season. Regarding seasons, spring was defined as from March to May, summer from June to August, autumn from September to November, and winter from December to February. The differences of injured workers between seasons were analyzed by *chi-square* test. As the weather and air pollution concentrations data on case days and control days were tested to be normally distributed, we described them with the mean and standard deviation, respectively, and compared the differences between them using *t*-tests. Next, the odds ratio of injury for a 10-mm increase in precipitation and its 95% confidence interval (*CI*) were calculated using a conditional logistic regression model:


$$\begin{array}{l} \log\left(Y\right)=\text{α}+\;{\text{β}}_{1}\left(precipitation_{t}\right)+{\text{β}}_{2}\left({wind\;speed}_{t}\right)\\ \;+\;ns\left({temperature}_{t},\;3\right)+{\text{β}}_{3}\left({sunlight}_{t}\right)+{\text{β}}_{4}\left(PM_{2.5t}\right)\\ \;+{\text{β}}_{5}\left(NO_{2t}\right)+{\text{β}}_{6}\left(SO_{2t}\right)+Holiday\;+\text{ strata}\left(ID\right) \end{array}$$

Where *t* is the day of lag (*t* = 0, 1, 2, 3). In this study, we only considered up to 3 days of lag as literature since injuries were more likely to occur within a short period following precipitation (29). *Y* denotes a case day or a control day (0 for control and 1 for case); *ns* (…) is a natural cubic spline, with 3 *dfs* to fit the nonlinear impact of temperature. The *df* was chosen by the lowest Akaike's Information Criterion for quasi-Poisson (*quasi-AIC*). The holiday is a categorical variable indicating a public holiday (0 for working days, 1 for holidays).

Based on the model, we estimated the excess rate of work-related injury for each 10mm increment in precipitation. The excess rate (*ER*) was computed as (odds ratio – 1.0]) ^*^ 100%. *ER* was statistically significant at the alpha=0.05 level if its 95% confidence interval (*CI*) did not cover zero. Furthermore, we performed stratified analyses by sociodemographic information, the feature of injury and clinical information.

Furthermore, a sensitivity analysis was performed to confirm the robustness and reliability of the estimates of excess rates, and to assess whether the general results were influenced by some specific participants. In the sensitivity analysis, we excluded the participants who were injured at school or public place and re-run all the models.

All statistical analyses were performed in R 4.1.1, primarily using the “rSPARCS” and “survival” packages (28). This study was approved by the Institutional Review Board (L2022-056) at Sun Yat-sen University.

## 3. Results

### 3.1. Baseline characteristics and meteorological conditions

Table 1 shows the descriptive information and seasonal distribution of injured workers reported by the surveillance-based database. A total of 28,332 cases were included, of which the largest proportion was reported in summer (32.6%, *n* = 9,241), and the smallest proportion in winter (16.3%; *n* = 4,607). Overall, the percentage of injured workers was higher among males, economically productive age groups (18–44 years old) and those with lower educational levels. As observed in Table 1, the number of work-related injuries decreased over the years across all seasons. Concerning the features of injuries at work, the most common cause was penetrating injury (41.4%) and the highest percentage of work-related injuries was recorded in industrial or building sites (72.5%). By clinical information on the injuries at work, the most common category was penetrating or open wounds (48.7%) and the body part involved was primarily upper extremities (52.4%). The number of workers who were injured moderately (91.4%, *n* = 25,906) is eleven times higher than that of severely (8.6%, *n* = 2425). In terms of the system involved, most workers ended up with movement system injuries (91.8%), while only 8.2% of cases suffered non-movement-system injuries.

**Table 1:** Characteristics of participants eligible for work-related injuries at injury sentinel monitoring hospitals in Guangzhou, China, by season 2016–2020 (*n* = 28,540).

**Factors**	**Total *N* (%)**	**Spring *N* (%)**	**Summer *N* (%)**	**Autumn *N* (%)**	**Winter *N* (%)**	***P*-value**
**Overall**	28,332	6,788	9,241	7,696	4,607	
**Sex**						
Male	23,761 (83.9)	5,671 (83.5)	7,822 (84.6)	6,434 (83.6)	3,834 (83.2)	0.091
Female	4,571 (16.1)	1,117 (16.5)	1,419 (15.4)	1,262 (16.4)	773 (16.8)	
**Age**						
18–44	18,493 (67.2)	4,588 (69.5)	5,963 (66.5)	4,961 (66.4)	2,981 (66.4)	<0.001
45–64	9,038 (32.8)	2,015 (30.5)	3,005 (33.5)	2,512 (33.6)	1,506 (33.6)	
**Education**						
Middle school and below	18,764 (66.2)	4,495 (66.2)	6,267 (67.8)	5,048 (65.6)	2,954 (64.1)	<0.001
High school and above	9,566 (33.8)	2,292 (33.8)	2,973 (32.2)	2,648 (34.4)	1,653 (35.9)	
**Occupation**						
Agricultural/technical worker	4,404 (15.8)	985 (14.7)	1,437 (15.8)	1,215 (16.1)	767 (17.0)	<0.001
Business/service worker	5,917 (21.2)	1,205 (18.0)	1,948 (21.4)	1,754 (23.2)	1,010 (22.4)	
Production and transportation equipment operator	17,560 (63.0)	4,513 (67.3)	5,711 (62.8)	4,594 (60.7)	2,742 (60.7)	
**Year**						
2016	6,032 (21.3)	1,466 (21.6)	1,961 (21.2)	1,676 (21.8)	929 (20.2)	<0.001
2017	5,796 (20.5)	1,655 (24.4)	1,879 (20.3)	1,442 (18.7)	820 (17.8)	
2018	6,055 (21.4)	1,429 (21.1)	1,823 (19.7)	1,671 (21.7)	1,132 (24.6)	
2019	5,410 (19.1)	1,198 (17.6)	1,710 (18.5)	1,477 (19.2)	1,025 (22.2)	
2020	5,039 (17.8)	1,040 (15.3)	1,868 (20.2)	1,430 (18.6)	701 (15.2)	
**Alcohol use**						
Used	110 (0.4)	34 (0.5)	23 (0.2)	24 (0.3)	29 (0.6)	0.002
Unused	28,213 (99.6)	6,751 (99.5)	9,217 (99.8)	7,671 (99.7)	4,574 (99.4)	
**Household register**						
Provincial domicile	5,885 (20.8)	1,424 (21.1)	1,816 (19.7)	1,663 (21.6)	982 (21.4)	<0.001
City domicile	6,337 (22.4)	1,277 (18.9)	2,305 (25.0)	1,673 (21.8)	1,082 (23.6)	
Out of province	16,053 (56.8)	4,063 (60.1)	5,112 (55.4)	4,352 (56.6)	2,526 (55.0)	
**Cause of injury**						
Traffic accident	438 (1.5)	95 (1.4)	144 (1.6)	124 (1.6)	75 (1.6)	<0.001
Falls	5,266 (18.6)	1,281 (18.9)	1,576 (17.1)	1,469 (19.1)	940 (20.4)	
Blunt injury	10,536 (37.2)	2524 (37.2)	3,308 (35.8)	2,906 (37.8)	1,798 (39.0)	
Sprain	364 (1.3)	97 (1.4)	96 (1.0)	102 (1.3)	69 (1.5)	
Penetrating injury	11,728 (41.4)	2,791 (41.1)	4,117 (44.6)	3,095 (40.2)	1,725 (37.4)	
**Location of injury**						
Industrial/building site	20,551 (72.5)	5,106 (75.2)	6,682 (72.3)	5,492 (71.4)	3,271 (71.0)	<0.001
Roads/street	488 (1.7)	80 (1.2)	180 (1.9)	142 (1.8)	86 (1.9)	
Trade and service venue	5,520 (19.5)	1,272 (18.7)	1,729 (18.7)	1,562 (20.3)	957 (20.8)	
Farm/farmland	468 (1.7)	121 (1.8)	138 (1.5)	124 (1.6)	85 (1.8)	
School/public place	1,303 (4.6)	208 (3.1)	511 (5.5)	376 (4.9)	208 (4.5)	
Glass/ceramic/mineral product	1,672 (7.4)	313 (5.8)	706 (9.5)	487 (7.9)	166 (4.5)	<0.001
Building/ground/obstacle	4,241 (18.7)	1,038 (19.2)	1,238 (16.6)	1,179 (19.2)	786 (21.3)	
Metal product	10,423 (45.9)	2,443 (45.1)	3,537 (47.4)	2,837 (46.2)	1,606 (43.4)	
Equipment	6,033 (26.5)	1,551 (28.6)	1,871 (25.1)	1,548 (25.2)	1,063 (28.8)	
Biology	210 (0.9)	36 (0.7)	68 (0.9)	62 (1.0)	44 (1.2)	
Plastic/rubber product	147 (0.6)	39 (0.7)	46 (0.6)	30 (0.5)	32 (0.9)	
**Category of injury**						
Contusion/sprain/strain	11,168 (39.5)	2,896 (42.7)	3,247 (35.2)	3,057 (39.8)	1,968 (42.8)	<0.001
Fracture	2,791 (9.9)	701 (10.3)	833 (9.0)	744 (9.7)	513 (11.2)	
Brain/internal organ injury	555 (2.0)	138 (2.0)	145 (1.6)	157 (2.0)	115 (2.5)	
Penetrating/open wound	13,781 (48.7)	3,046 (44.9)	5,006 (54.2)	3,726 (48.5)	2,003 (43.6)	
**Affected body part**						
Multi-part	881 (3.1)	210 (3.1)	282 (3.1)	246 (3.2)	143 (3.1)	<0.001
Torso	2,587 (9.1)	617 (9.1)	780 (8.4)	739 (9.6)	451 (9.8)	
Upper extremities	14,838 (52.4)	3,643 (53.7)	4,816 (52.1)	3,885 (50.5)	2,494 (54.1)	
Head	3,588 (12.7)	858 (12.6)	1,088 (11.8)	1,010 (13.1)	632 (13.7)	
Lower extremities	6,436 (22.7)	1,460 (21.5)	2,274 (24.6)	1,815 (23.6)	887 (19.3)	
**Severity**						
moderate	25,906 (91.4)	6,206 (91.4)	8,459 (91.5)	7,048 (91.6)	4,193 (91.0)	0.705
severe	2,425 (8.6)	582 (8.6)	781 (8.5)	648 (8.4)	414 (9.0)	
**System involved**						
Non-movement system	2,314 (8.2)	601 (8.9)	674 (7.3)	534 (6.9)	505 (11.0)	<0.001
Movement system	26,014 (91.8)	6,184 (91.1)	8,566 (92.7)	7,162 (93.1)	4,102 (89.0)	

The difference of work-related injuries between seasons was tested by Chi-square. P < 0.05 indicated that there was statistically significant difference in work-related injuries between seasons.

Precipitation and air pollution levels on case days and control days for the entire year during the study period are presented in Table 2. We found the average daily precipitation on case days (7.08 mm) was significantly higher than that on control days (6.71 mm), whereas the wind speed, sunlight and temperature did not.

**Table 2:** Distribution of weather and pollutant concentrations for case days and control days in Guangzhou, China, 2016–2020.

	**Control days [mean (SD)]**	**Case days [mean (SD)]**	***P*-value**
Number	96,769	28,332	
Wind speed (m/s)	2.21 (1.00)	2.20 (0.98)	0.316
Sunlight (h)	4.60 (3.77)	4.61 (3.76)	0.717
Precipitation (mm)	6.71 (17.93)	7.08 (18.68)	0.002
Temperature (°C)	23.55 (5.62)	23.55 (5.62)	0.895
NO_2_ (μg/m^3^)	34.10 (20.07)	34.64 (20.57)	<0.001
PM_2.5_ (μg/m^3^)	29.00 (18.05)	29.31 (18.50)	0.012
SO_2_ (μg/m^3^)	9.37 (4.30)	9.46 (4.38)	0.003

SD, Standard deviation. P < 0.05 indicated statistically significant difference in weather and pollutant concentrations between case days and control days.

### 3.2. Major effects

Table 3 further shows the effect of precipitation on injuries at work over lag 0–3 days. We found that each 10 mm increase in precipitation at lag0 was associated with a 1.61% (95%*CI*: 0.82–2.40%) increase in the risk of work-related injuries. The excess risk of injury gradually decreased over lag days, to 1.46% (95%*CI*: 0,69–2.24%) at lag1, 1.39% (0.61–2.17%) at lag2 and 1.18%(0.38–1.99%) at lag 3.

**Table 3:** Excess rate of work-related injuries associated with 10 mm increase in precipitation, by lag time.

**Lag days**	***N* case**	**Excess Rate % (95% *CI*)**	***P*-value**
0	28,010	1.61 (0.82,2.40)	<0.001
1	28,010	1.46 (0.69,2.24)	<0.001
2	28,026	1.39 (0.61,2.17)	<0.001
3	28,039	1.18 (0.38,1.99)	0.004

CI, confidence interval.

### 3.3. Stratification effects

**Figure 1:**
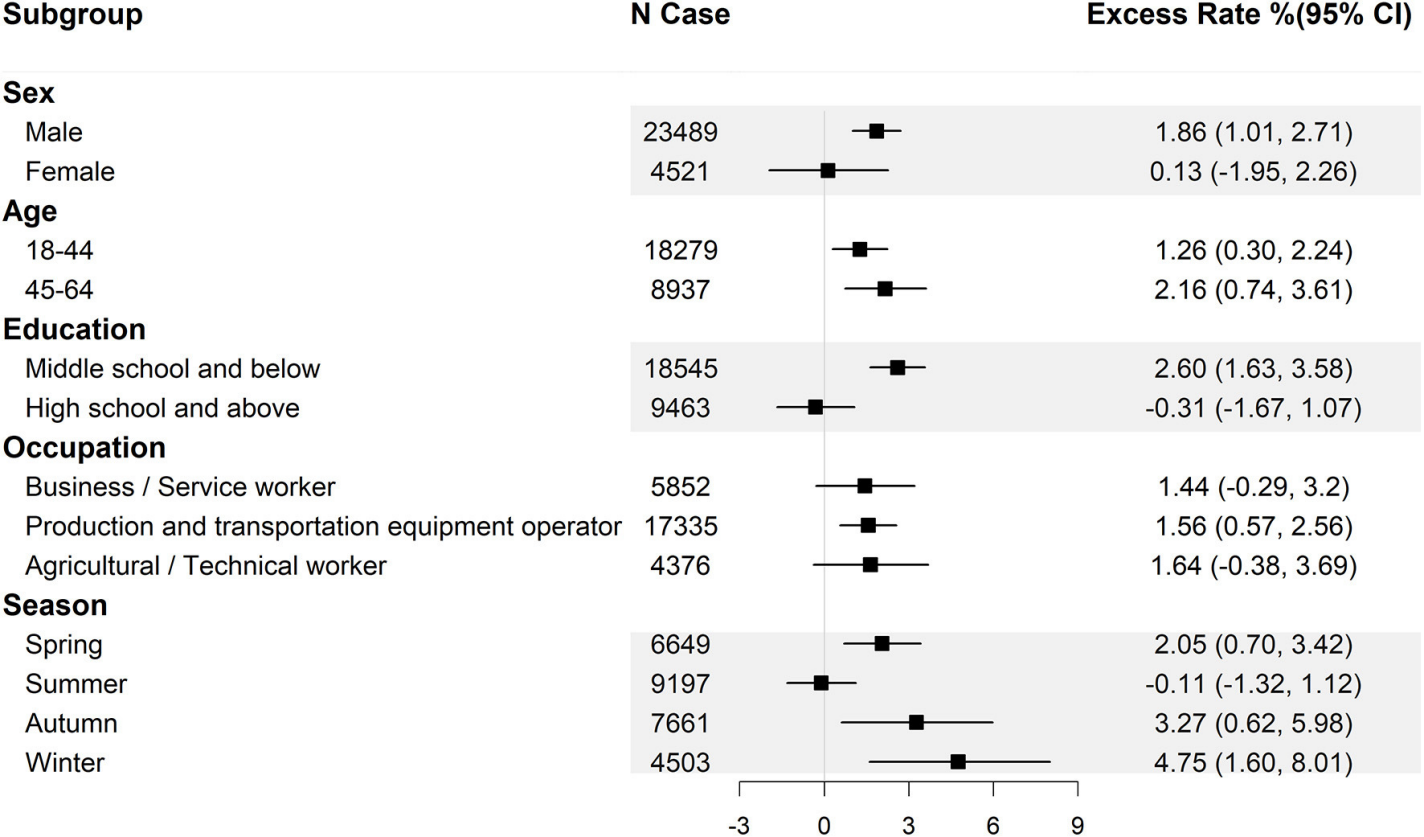
Excess rate (and 95% confidence intervals) of injuries at work associated with 10 mm increase in precipitation by district, sex, age, education, occupation, and season. CI, confidence interval.

**Figure 2:**
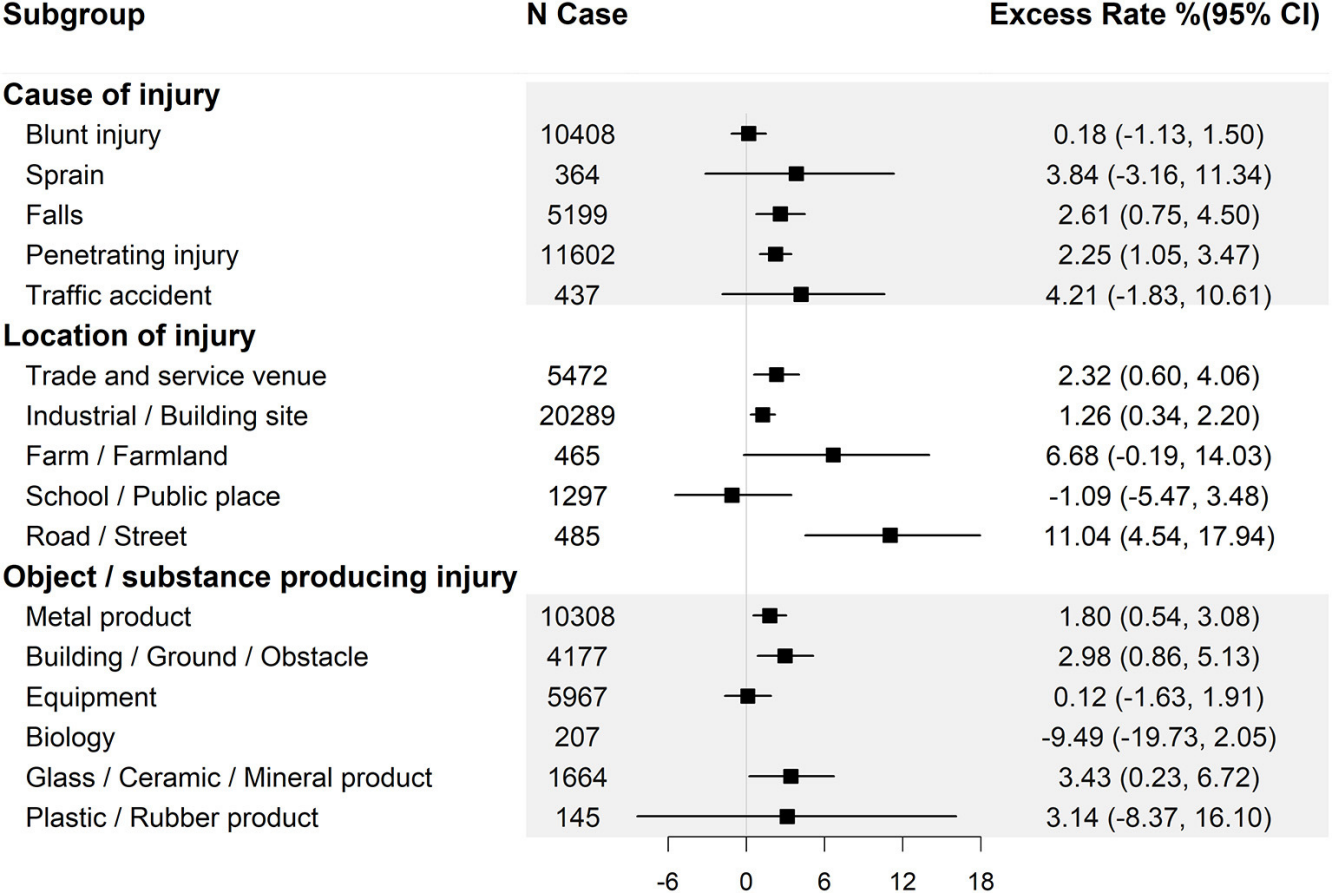
Excess rate (and 95% confidence intervals) of injuries at work associated with 10 mm increase in precipitation by cause of injury, location of injury, and item involved. CI, confidence interval.

**Figure 3:**
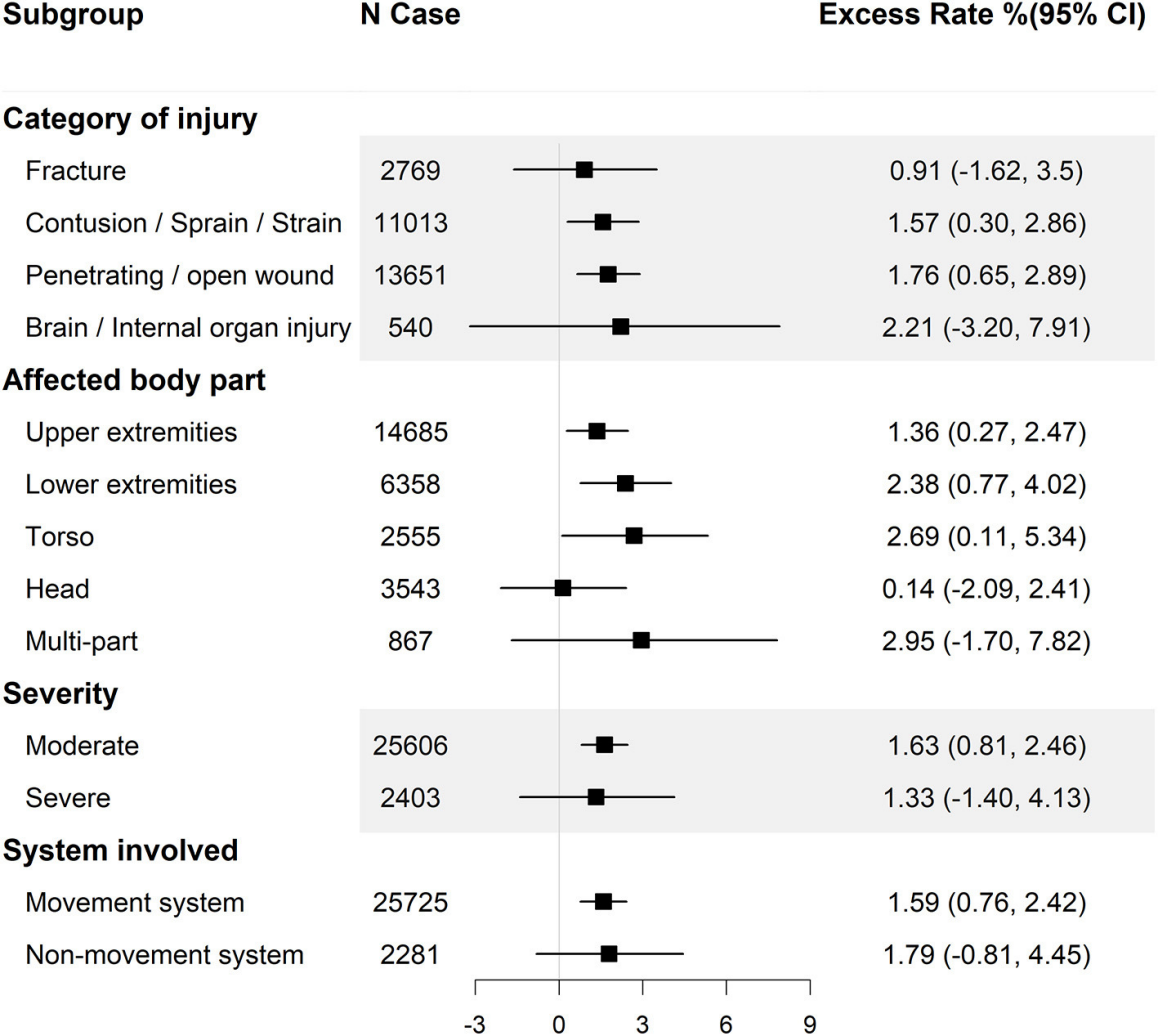
Excess rate (and 95% confidence intervals) of injuries at work associated with 10 mm increase in precipitation by nature of injury, injury area, severity, and system involved. CI, confidence interval.

Since precipitation on the day of injury (lag 0) has the highest excess rate, we stratified the effect estimates on lag 0 by basic information (Figure 1), the features of injury (Figure 2) and clinical information (Figure 3).

When the results were stratified by basic information, we found higher effect estimates among the males and those aged 45–64 years, compared to among the females and among those below 45. We found significant effect estimates among the participants with middle school and below education but not among those with high school and above education. Furthermore, the effects estimates stratified by occupation indicated that the precipitation had a significantly adverse effect on production and transportation equipment operators but had no significant effect on other work types. In terms of seasons, the highest effect estimate was recorded in winter with an excess rate of 4.75% (95%*CI*: 1.60–8.01%), followed by autumn and spring, whereas there was no significant effect in summer.

Similarly, when the results were stratified by the features of the injury, we found a 10 mm increase in precipitation was significantly associated with the work-related injuries caused by falls and penetrating injuries, but not those caused by sprains or blunt injuries (Figure 2). We also found the effects estimates were significant when the injury occurred on roads, trade venues and industrial sites, but not in other places such as farm and school (Figure 2). Furthermore, injuries at work related to glass, ceramic products, buildings, obstacles and metal products were statistically associated with precipitation, but those related to equipment, biology, plastic or rubber products did not.

In addition, when stratifying the results according to clinical information, we found that the work-related injuries where the categories of injuries belonged to open wounds, as well as sprains were significantly associated with precipitation, while other categories of injuries such as fracture and internal organ did not (Figure 3). In terms of the affected body part, we found the work-related injuries affected the torso, lower extremities and upper extremities were more related with precipitation, whereas the injuries affected head and multi-part did not (Figure 3). In the sensitivity analyses with the removal of workers injured at school or public place, excess rate estimates from this model were robust, indicating a stable relationship between precipitation and injuries during work.

## 4. Discussion

Measuring, understanding, and intervening the risk factors of work-related injuries should be considered a foundational component of worker health research. The current study revealed a positive effect of precipitation on work-related injuries and extended the prior studies by capturing the accurate and complete details of injured workers ranging from sociodemographic information to the features of injury to clinical information. Collectively, these findings can help identify which subgroups of workers are more sensitive to injuries on rainy days. It also helps discover which type of occupational injuries are more likely to occur on rainy days. Public agencies may be able to use the results to announce precise safety precautions regarding injuries to sensitive workers.

The availability of information concerning the effect of precipitation on work-related injuries remains limited, so we were unable to compare the findings with the available evidence. Yet, a few studies have observed the association between precipitation and total injuries. For instance, a 4-year retrospective study in Korea found that the relative risk of injuries increased as the amount of precipitation increased (16). Another study in the United States also reported that total injury incidence was positively associated with the amount of precipitation (17). In this study, the estimated excess rate provided a new insight that the precipitation positively affected work-related injuries. There are some possible explanations that rainfall may create a water film on the surface of workplaces such as pavements and high constructions, causing a decrease in skid resistance, which may contribute to an increased likelihood of slipping on the roads and falling from high (30). There might be another possibility that wearing raincoats, taking an umbrella and rain itself may increase the difficulty of work by narrowing the visual fields or making the working environment slippery (31, 32).

We found a significant lagged effect of precipitation on work-related injuries, especially in the following 3 days, indicating that weather conditions in the past 3 days should be considered for worker protection. A few previous studies observed a similar lagged effect of precipitation on total injuries. For example, a study in Korea found that precipitation with an amount of 250 mm or more affected the risk of injuries over the following 2 days (16). This study also found that the lagged effect of precipitation on work-related injuries gradually diminished over time. This finding was consistent with the Korean study, which showed that the effect of precipitation on injury risk decreased over time (16). The lagged effect observed in our study may be explained by that the slippery working conditions may continue for a few days after the rain. Therefore improving ventilation systems in workplaces would mitigate the impact.

We found that males and the elderly (45–64 years old) workers were more vulnerable to injuries associated with precipitation, suggesting that promoting preventive strategies among these sensitive workers against the health risk of rainy days might reduce the injury rate. Previous studies have reported biological changes in sensory, cognitive, and physical abilities and aging can affect the capability of workers (33), and one study conducted by Omranian et al. reported a higher injury risk on rainy days for the older drivers (31). Another study in Brunei Darussalam found male workers had a disproportionately higher risk of suffering nonfatal occupational injuries than female workers (11). There are some possible explanations that males are more likely to be employed in labor-intensive and high-risk industries such as construction and transportation, where workers are more likely to get injured on rainy days (11). And we speculated that elder workers are more sensitive to injuries in wet weather due to their decreased physical functions such as the reduced muscle strength, standing balance, visual acuity, range of motion of lower extremities and the increased reaction time, which may, in turn, alter the quality of work performance and the ability to notice work environment hazards (34, 35).

Moreover, we found workers with lower educational levels were more sensitive to the occurrence of injuries during rainy periods. This result was supported by most occupational health and safety studies, which revealed that increased educational level was associated with decreased work-related injuries (36, 37). The result was explained by that more educated people were less likely to get injured at work as they tended to have better access to information regarding hazardous or risky jobs (38). There might be another possibility that more educated workers might have equivalent intelligent ability to avoid injuries in the rain by figuring out the locations of the risky materials or objects at workplaces in advance, or holding more cautious work attitudes and behaviors (39).

Production equipment operators or transport workers were observed to have a higher risk of injuries on rainy days. As an important weather parameter, precipitation has been frequently considered in road traffic injury studies, and some findings suggested that precipitation had non-significant or even negative effects on road traffic injuries (40). In general, the findings were explained by the possible reasons that rainfall increased the caution of drivers and decreased the number of pedestrians and traffic activity (32). However, not all injuries can be prevented by driving carefully, and some other studies have suggested that rainfall is positively associated with road traffic injury (19, 30, 41). Some possible explanations are that when drivers work in wet weather, the rain may reduce the friction between pavement and tires, increase the difficulty of device handling, and decrease device performance simultaneously (32). Preventive measures such as road safety measures and safer tools may help reduce the incidence of injury among production equipment operators or transport workers.

The study highlighted a greater risk of work-related injuries on rainy days in winter rather than in other seasons. A few studies have compared the effect of precipitation on specific injuries across seasons (42, 43). For example, one study conducted in Finland found that the number of fractures was 2.5 times greater on slippery winter days and 1.4 times greater on normal winter days, compared to non-winter days (21). The findings can be explained that, in winter, workers were more frequently exposed to cold temperatures, had slower reaction times, and lost more bone mass, resulting in more fractures (43).

This study demonstrated that precipitation was a risk factor for work-related injuries caused by falls and penetrating injuries. A previous study in Brunei Darussalam reported that the three leading causes of occupational injuries were struck by falling objects, falls from height, and contact with sharp items (11). We also found that work-related injuries that occurred on construction sites, trade venues, or roads were significantly associated with precipitation. The reports from different countries suggested that occupational injuries were a major concern for high-risk work sectors such as construction and manufacturing (44, 45). Furthermore, the results in our study proved that precipitation was a crucial factor for work-related injuries which affected the torso or extremities. Reports from the Brunei and Ireland found that upper and lower extremities were the commonest body part involved in work-related injuries (9, 11). These findings can be explained by workers being more likely to have falls, which commonly resulted in limb injuries (46), as the rain reduced the friction between the pavement and shoes or flooded the roads. In short, this study explains the findings from the other studies cited and allows our results to be more transferable to public health and prevention domains by estimating work-related injury risk attributable to precipitation.

This study has several strengths. Overall, this is one of the first studies exploring the association between precipitation and work-related injury. Secondly, the sentinel-surveillance injury data used in this study contains a more comprehensive set of injuries than admission only, reflecting a broader population with injuries at work. Furthermore, the adequate stratification analysis provides strong evidence for accurate prevention and control of work-related injuries. However, there are also some limitations to this study. First, observational data on air pollution and weather were limited. Therefore, cases had the same nearest monitoring site were assigned the same observations. However, this limitation is likely a combination of Berkson and classical error (47), resulting in a bias toward the null and underestimate of the precipitation effect. Second, similar to previous studies (48–50), we did not distinguish the impact on indoor and outdoor workers due to the lack of information on the indoor injuries or how it was associated with the outdoor precipitation. Future studies may complement the findings of this study by collecting the data of indoor injuries. Third, this study may be prone to unmeasured (or residual) confounding issues and exposure misclassification issues which are common among observational and ecological studies.

## 5. Conclusion

In summary, an increase in precipitation was associated with a relatively high prevalence of work-related injuries. It is worth noting that male and elderly workers, as well as those with lower educational levels, were more vulnerable to injuries on rainy days. Findings from this study can help the health sectors and relevant stakeholders to better prevent work-related injuries caused by rainfall. The analysis strategies and framework undertaken in the current study may also be generalized to other regions, further aiding in the efforts to improve workers' safety and health conditions and reduce the overall disease burden of work-related injuries.

## Data availability statement

The data that has been used is confidential. Requests to access the datasets should be directed to YH, haoyt@bjmu.edu.cn.

## Ethics statement

The studies involving human participants were reviewed and approved by Sun Yat-sen University Institutional Human Ethics Committee (Ethics Approval Number: SYSU-SPH-2020126). Written informed consent for participation was not required for this study in accordance with the national legislation and the institutional requirements.

## Author contributions

TT, XL, and TH wrote the main manuscript text and analyzed the data. CS, PW, SC, TG, ZL, and PQ cleaned the data and prepared Figures 1–3. KZ revised the article. BL, WZ, and YH collected the data and provided methodological guidance. All authors reviewed the manuscript. All authors contributed to the article and approved the submitted version.
